# HR-pQCT imaging in children, adolescents and young adults: Systematic review and subgroup meta-analysis of normative data

**DOI:** 10.1371/journal.pone.0225663

**Published:** 2019-12-13

**Authors:** Daddy Mata-Mbemba, Taryn Rohringer, Ala Ibrahim, Thomasin Adams-Webberc, Rahim Moineddin, Andrea S. Doria, Reza Vali

**Affiliations:** 1 Department of Diagnostic Imaging, IWK Health Centre, and Department of Diagnostic Radiology, Dalhousie University, Halifax, Nova Scotia, Canada; 2 Department of Diagnostic Imaging, Hospital for Sick Children and Department of Medical Imaging, University of Toronto, Toronto, Canada; 3 University of Toronto, Toronto, Canada; 4 Hospital Library and Archives (T.A.W.), Hospital for Sick Children, Toronto, Canada; 5 Departments of Family and Community Medicine (R.M.), University of Toronto, Toronto, Canada; University of Mississippi Medical Center, UNITED STATES

## Abstract

We aimed to investigate the methodologies on image acquisition of normative data of high-resolution peripheral quantitative computed tomography (HR-pQCT) in children, adolescents and/or young adults (up to 25 years) and to determine their normative data based on available literature. A literature search was conducted in MEDLINE, EMBASE and Web of Science from 1947 to July 2019. Quality of articles was assessed using Standards for Reporting of Diagnostic Accuracy (STARD) scoring system and Modified Newcastle-Ottawa scale (NOS). Articles which fitted the following criteria were combined to meta-analysis: age range (15 to 22.6 years), references at tibia (22.5mm) and/or radius (9.0 to 9.5mm). Eight articles were ultimately included in the systematic review and 4 of them that filled the criteria were summarised in meta-analysis. The results of random effects model of HR-pQCT parameters of the 4 articles were as follows: 1)Radius: bone volume fraction (BT/BV) [estimate 0.17:0.1229(lower)-0.2115 (upper); trabecular number (Tb_N):2.08(2.03–2.12); trabecular thickness (Tb.Th):0.07 (0.07–0.0.08); trabecular separation (Tb.Sp):0.41 (0.38–0.42); cortical thickness (Ct.Th):0.85 (0.76–0.94); cortical porosity (Ct.Po):1.53 (0.63–2.44); total area (Tt.Ar):263.66(-385.3–912.6); total bone density (Tt-vBMD):280.5 (73.1–487.7); Trabecular density (Tb-vBMD):223.6 (47.1–400.09), and cortical density (CT.vBMD):765.9 (389.1–1142.8). 2)Tibia: BT/BV:0.18 (0.17–0.19); Tb_N:2.02 (1.83–2.2); Tb.Th:0.08 (0.80–0.09); Tb.Sp:0.40(0.36–0.44); Ct.Th:1.32(1.26–1.38); Ct.Po:3.15 (1.1–5.2); Tt.Ar:693.1(150.2–1235.8); Tt-vBMD:343.76 (335.5–352.1); Tb-vBMD:223.6 (213.37 (193.5–233.2), and CT.vBMD:894.3 (857.6–931.1). There is overall ‘fair’ evidence on reporting of results of normative data of HR-pQCT parameters in children, adolescents and/or young adults. However, data are scarce pointing out to the urgent need for standardization of acquisition parameters and guidelines on the use of HR-PQCT in these populations.

## Introduction

Bone strength, a critical measure of skeletal health and fracture risk, is a composite of bone density and bone quality. The current gold standard imaging technique for assessing skeletal fragility is Dual Energy X-ray Absorptiometry (DXA), which calculates areal bone mineral density (BMD). DXA uses bone density as a marker for bone strength, but lacks insight into bone quality parameters that may significantly alter the patient’s bone health [1,2]. A more detailed analysis of bone microarchitecture may be achieved through bone biopsy, but such a technique is invasive and therefore less desirable, especially for serial monitoring [3]. High resolution peripheral quantitative computed tomography (HR-pQCT) is a three-dimensional imaging technology that uses parallel CT slices captured at the distal tibia and/or radius to provide a volumetric, as opposed to areal, BMD in addition to various micro-architectural parameters for both trabecular and cortical bone [4,5]. HR-pQCT is non-invasive, but still allows for detailed assessment of both bone density and bone quality in its estimation of bone strength [6–11].

Bone development and achievement of robust bone strength are critical aspects of childhood and adolescent development [6]. By virtue of its two-dimensional measurement of BMD, DXA use is further limited in a pediatric population. The exclusion of bone depth in its measurement and lack of adjustment for patient size results in an under-estimation of bone density in smaller children and an over-estimation of bone density in larger children [2]. Such a limitation is circumvented by the volumetric BMD measurement with HR-PQCT. Additionally, HR-pQCT has a very low dose of ionizing radiation (3μSv per scan), which is comparable to the dose from a DXA scan (1–6 μSv per scan) [12]. The low dose of radiation with HR-pQCT scans enhances its utility in a pediatric population where substantial radiation, especially of epiphyseal growth plates, is to be avoided. Further, the invasiveness of bone biopsy renders its use further limited in a pediatric population. HR-pQCT has therefore emerged as an attractive imaging option for assessing skeletal strength in younger patients. This is reinforced by the growing body of literature using HR-pQCT to assess bone parameters as an index of bone strength in disease, treatment response and clinical fracture risk in children [1,2,6,13].

One major barrier that remains in both the research and clinical application of HR-pQCT is the lack of standardized normative values for the micro-architectural and volumetric BMD parameters. This gap in the literature with respect to standardized reference values, as is used in calculation of Z-scores in conjunction with DXA imaging, is notably lacking for a pediatric population [6,14].

We aimed to investigate the various methodologies that exist for HR-pQCT image acquisition in children, adolescents and/or young adults (up to 25 years), including the region of interest (ROI) and site of acquisition, and to determine normative data in these age ranges, in order to direct guidelines that enable standardization for HR-pQCT in young patients. This will be accomplished through a systematic review and meta-analysis of published data with regards to HR-pQCT. This study endeavors to determine whether an aggregation of normative values in a pediatric population (aged 0–25 years old) is possible via synthesis of the literature, as well as whether any associations exist between HR-pQCT parameters in a healthy population of this age and clinical/laboratory parameters and bone health values from other imaging modalities.

## Materials and methods

This systematic review and subgroup meta-analysis complied with the Preferred Reporting Items for Systematic reviews and Meta-Analysis guidelines [15]. Our institution’s research ethics board waived approval for secondary data acquisition from previously published papers available in the public domain.

### Literature search

**Fig 1:**
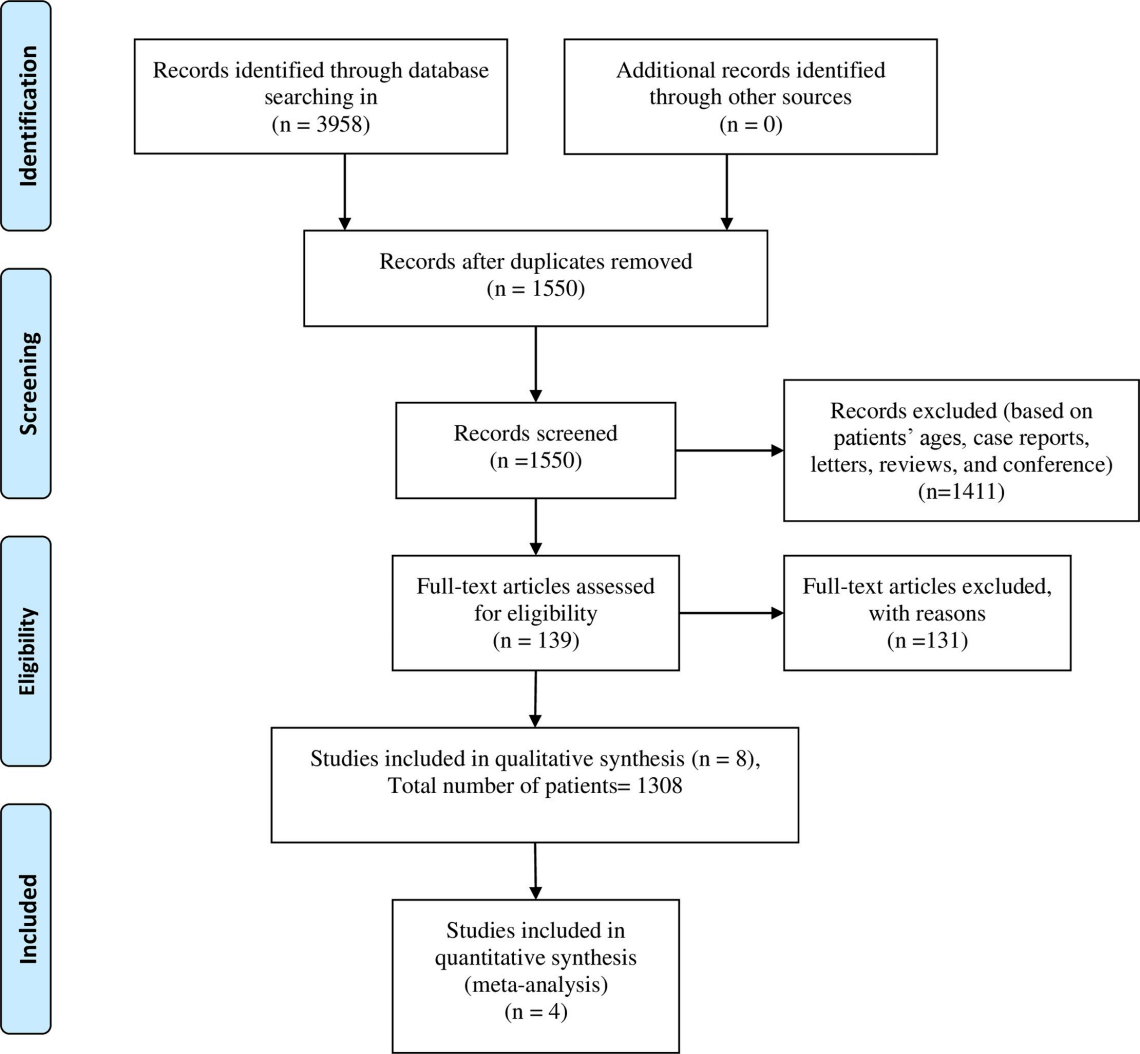
Flowchart of inclusion of papers in the study.

The databases Ovid MEDLINE Epub Ahead of Print, In-Process & Other Non-Indexed Citations, Ovid MEDLINE Daily, and Ovid MEDLINE (1946 to July 2019) and EMBASE Classic + Embase < 1947 to 2019 Week 30 > were searched to examine the use of HR-pQCT in normal children, adolescent and young adults. The search strategy was developed in collaboration with an experienced hospital librarian (T.A.W) and conducted by a radiologist (D.M.M). It included database subject headings (e.g. MeSH) and text words as follows: high resolution peripheral quantitative computed tomography, HR-pQCT, children, adolescents, adults. Studies were first screened by examining their titles and abstracts (D.M.M & T.A.V). The full texts of potentially eligible studies were retrieved for further review. No language restriction was applied. A manual search of additional records and reference lists was not performed. Fig 1 (following Prisma recommendation [16]) as well as S1 Appendix contain the search strategies.

### Article inclusion and exclusion criteria

The following inclusion criteria were used for this systematic review: a) Study aiming at evaluating the distal tibia and/or radius of normal subjects using HR-pQCT. Studies evaluating diseases or changes after intervention were included if the baseline data of normal subjects or data of control normal groups could be extracted separately; b) The paper provided data related to structural parameters and/or bone densities parameters provided by HR-pQCT. c) The paper included children, adolescents and/or young adults with ages up to 25 years. If both children/adolescents and adults were included, data on children, adolescents would have to be separately extractable. d) If the patient population of one article overlapped with the patient population of another article, the article with the larger sample size would be included. Case reports, case series, review articles, pictorial essays, letters to editors, unpublished data, conference abstracts, and proceedings on the topic of interest were excluded.

Afterwards, 4 articles [13,17–19] which evaluated adolescent and young adults of similar age range (15 to 22.6 years) using the same references at the tibia (22.5 mm) and/or radius (9.0 to 9.5 mm) were combined into a meta-analysis to summarize their data. In all these papers, authors used the same HR-pQCT scanner (XtremeCT I; Scanco Medical, Switzerland). No study used XtremeCT II.

### Data extraction

One reader (D.M.M) reviewed the full text of candidate articles and selected those that met the inclusion criteria. A second reader (R.V) reviewed the process for inclusion of articles in both the systematic review and meta-analysis. There were no inter-reader disagreements (Kappa coefficient = 1.0).

Data extracted included the following: study characteristics, patient demographic information; HR-pQCT scanning references, and information regarding HR-pQCT structural and density parameters at tibia and/or radius, as shown in the Tables 1–3.

**Table 1:** Demographic characteristics of patients and technical HR-PQCT information.

First author's last name/ publication year	Number of patients (n)	Patient population	Gender	Age range (years)	Age mean (years)	Pubertal status	Mean Height	Mean Weight	Mean BMI	ROI (Tibia, Radius or both) and offset types
Cheuk 2016	52	pediatric	Male & female	13–16	Boy: 14.1 ± 1.02 Girl: 15.05 ± 1.24	Boy: 4.0(2.5–5.0) Girl: 4.0(2.5–5.0)	Boy: 164.9(157.7–170.4) Girl: 157.5 (153.7–161.9)	Boy: 54.2(45.4–58.4) Girl: 44.6(42.6–47.6)	Boy: 19.3±3.1 Girl: 18.4 ±1.7	Radius, %offset (5% versus 4%)
Ackerman 2011	34	Pediatric and young adult	female	15–21	EA: 18.7 ± 1.7 NC: 19.4 ± 1.2	EA//:17.5± 0.9 NC//:17.7±0.9	EA:165.8 ± 7.8 NC:161.4 ± 7.6	N/AV	EA:22.2 ± 2.4 NC:21.4 ± 2.4	Radius & tibia, fixed-offset (radius 9 mm & tibia 22.5 mm)
Kawalilak 2017	32	pediatric	Male & female	8 to 13	Boy: 11.6 ±1.4. Girl:10.9±1.8	N/AV	Boy: 152.4 ± 10.3 Girl: 147.3 ± 14.0.	Boy: 44.8 ± 13.3 Girl: 39.2 ± 10.9.	N/AV	Radius & tibia, %offset (radius 7% and tibia 8%)
Gabel 2017	393	Pediatric and young adult	Male & female	9.5 to 20.3	Boy:15.1 ±2.6 Girl:14.5 ± 3.4	Boy§: 20/14/11/63/75 Girl§: 31/37/33/58/50	Boy:167.1±14.3 Girl:155.5±11.6	Boy: 60.8 ± 17.3 Girl: 50.1 ± 14.1	N/AV	Radius & tibia, %offset (radius 7% & tibia 8%)
Kirmani 2012	118	Pediatric and young adult	Male & female	6 to 21	N/AV	N/AV	N/AV	N/AV	N/AV	Radiusfixed-offset (radius: 9.02 mm)
Burt 2014	59	Pediatric and young adult	Male & female	16 to 19	Boy:18.7±1.0 Girl:18.7±1.0	N/AV	Boy: 1.76±0.1. Girl:1.64±0.1	Boy: 74.8±13.6. Girl: 62.7±10.0.	N/AV	Radius & tibia, fixed-offset (radius 9.5 mm & tibia 22.5 mm)
Chevaley 2017	152	Young adults	Male	22.6 years	22.6±0.8	Tanner 5	178.4±6.1	73.2±12.4	23.0±3.6	Radius & tibia, fixed-offset (radius 9.5 mm & tibia 22.5 mm)
Rudang 2013	292	Young adults	Male & female	18–20	18.9±0.6		181.6±6.8	78.2±12.1	N/AV	Radius & tibia, fixed-offset (radius 9.5 mm & tibia 22.5 mm)

Abbreviations

**†** EA: eumenorrheic athletes (EA)

‡NC: nonathletic controls

//: Bone age. N/Av: not available

§:Tanner 1/2/3/4/5, (n): number total of patients included in the study (male + female).

**Table 2:** HR-pQCT parameters used in the selected studies of this review: Radius.

First author's last name/ publication year	Age mean (years)	BV/Tv±SD	Tb.N±SD	Tb.Th±SD	Tb.sp±SD	Tb.1/N.SD±SD	Ct.Th±SD	Ct.po±SD	Ct.Po.Dm±SD	CT.ar±SD	Tt.ar±SD	Tt.vBMD±SD	Tb.vBMD±SD	Tb.Meta.vBMD±SD	Tb.Inn.VBMD±SD	CT.vBMD±SD
Cheuk 2016	5% Protocol	-	1.5±0.19	0.07±0.01	0.58±0.09	-	0.75 (0.57–0.92)	-	-	39.88±11.31	129.6 (119.13–141.90)	308.96±56.54	144.56±29.43	-	-	786.34±68.86
	4% Protocol	-	1.54±0.20	0.07±0.01	0.58±0.09	-	0.75(0.58–0.98)	-	-	40.81±11.93	124.1(114.55–140.73)	319.85±67.47	144.69±30.82	-	-	793.24±74.62
Ackerman 2011	EA: 18.7 ± 1.7	--	2.04±0.22	0.07±0.01	0.42±0.05	--	0.71±0.16	--	--	49.5±9.3	231.3±44.9	180.5±30.5	306.1±46.8	--	--	306.1±46.8
	NC: 19.4 ± 1.2	--	2.07±0.21	0.08±0.01	0.41±0.05	--	0.86±0.19	--	--	56.6±12.8	191.7±41.7	188.8±34.9	352.8±67.9	--	--	352.8±67.9
Kawalilak 2017	11.3±1.6	0.154±0.025	2.3±0.2	0.687±0.011	0.377±0.031	0.144±0.018	0.350±0.138	7.0±2.3	0.160±0.01	19.9±8.1	168±33.7	259.6±37.8	185.1±30.0	253.5±33.2	137.7±29.2	647.6±52.8
Gabel 2017	Boy:15.1 ±2.6	0.158±0.030	1.98±0.26	0.080±0.015	0.433±0.700	--	1.02±0.32	3.4±2.1	--	60.7±21.3	262.7±59.6	326.3±81.8	--	--	--	729.9±109.7
	Girl:14.5 ± 3.4	0.141±0.026	1.97±0.26	0.072±0.010	0.446±0.073	--	0.87±0.28	2.4±1.9	--	60.7±21.3	262.7±59.6	305.4±77.9	--	--	--	735.3±138.8
Kirmani 2012	Boy: --	0.16(0.14–0.18)	2.03(1.88–2.18)	0.078(0.072–0.084)	0.41(0.37–0.45)	--	0.50(0.33–0.74)	0.85(0.61–1.10)	--	--	--	--	--	--	--	676(624–718)
	Female: --	0.14(0.12–0.16)	1.96(1.79–2.11)	0.072(0.067–0.076)	0.44(0.39–0.49)	--	0.37(0.21–0.72)	0.60(0.38–0.85)	--	--	--	--	--	--	--	651(572–786)
Burt 2014	Boy:18.7±1.0	--	2.15±0.25	--	--	--	0.96±0.22	2.68±1.22	--	--	357.1±63.9	327.8±64.4	204.9±45.0	--	--	846.1±49.3
	Girl:18.7±1.0	--	2.00±0.18	--	--	--	0.90±0.13	1.03±0.58	--	--	281.5±57.7	317.4±50.0	169.8±41.4	--	--	922.6±40.0
Chevaley 2017	22.6±0.8	0.164±0.025	2.09±0.23	0.078±0.011	0.406±0.053	--	0.85±0.13	1.74±0.71	--	--	--	335±44	196±30	--	--	852±32
Rudang 2013	18.9±0.6	0.171±0.03	2.12±0.26	0.081±0.13	0.399±0.061	--	0.873±0.176	1.12±0.49	0.146±0.014	--	--	--	--	--	--	--

Abbreviations: BV/TV: trabecular bone volume to total volume fraction; Tb.N (1/mm): Trabecular number; Tb.Th: trabecular thickness (mm); Tb.Sp: trabecular separation (mm); Ct.Th: cortical thickness (mm); Ct.Po: cortical porosity (%), Ct.Ar: cortical area (mm^2^); Tt.Ar: total area (mm^2^); Ct.vBMD: cortical volumetric bone mineral density (mg/cm^3^); Tb.vBMD: trabecular volumetric bone mineral density (mg/cm^3^); and Tt.vBMD: total bone mineral density (mg/cm^3^).

**Table 3:** HR-pQCT parameters used in the selected studies of this review: Tibia.

First author's last name/ publication year	Age mean (years)	BV/Tv±SD	Tb.N±SD	Tb.Th±SD	Tb.sp±SD	Tb.1/N.SD±SD	Ct.Th±SD	Ct.po±SD	Ct.Po.Dm±SD	CT.ar±SD	Tt.ar±SD	Tt.vBMD±SD	Tb.vBMD±SD	Tb.Meta.vBMD±SD	Tb.Inn.VBMD±SD	CT.vBMD±SD
Cheuk 2016		n/a	n/a	n/a	n/a	n/a	n/a	n/a	n/a	n/a	n/a	n/a	n/a	n/a	n/a	n/a
Ackerman 2011	EA: 18.7 ± 1.7	--	2.04±0.20	0.09±0.01	0.41±0.04	--	1.27±0.18	--	--	130.5±17.9	708.4±107.8	337.8±45.3	213.1±29.2	--	--	876.6±36.4
	NC: 19.4 ± 1.2	--	1.97±0.25	0.09±0.02	0.43±0.06	--	1.30±0.26	--	--	120.2±20.1	585.3±117.0	353.9±68.6	202.6±34.2	--	--	902.4±805
Kawalilak 2017	11.3±1.6	0.154±0.0.1	2.0±0.3	0.779±0.113	0.420±0.583	0.176±36.3	0.548±0.215	7.7±2.4	0.160±8.7	53.2±20.7	589.3±99.2	241.1±33.1	188.9±24.8	252.1±31.1	145.9±23.0	694.0±42.1
Gabel 2017	Boy:15.1 ±2.6	0.165±0.025	1.90±0.27	0.088±0.014	0.450±0.076	--	1.20±0.37	5.6±2.4	--	119.0±36.4	749.9±133.2	294.4±59.6	--	--	--	748.7±88.5
	Girl:14.5 ± 3.4	0.154±0.025	1.81±0.26	0.086±0.014	0.477±0.076	--	1.03±0.31	3.8±2.1	--	92.8±27.3	624.9±90.5	281.7±60.3	--	--	--	773.8±112.6
Kirmani 2012	Boy: --	--	--	--	--	--	--	--	--	--	--	--	--	--	--	--
	Female: --	--	--	--	--	--	--	--	--	--	--	--	--	--	--	--
Burt 2014	Boy:18.7±1.0	--	1.87±0.27	--	--	--	1.36±0.29	3.47±1.40	--	--	832±155.9	341.2±55.4	221.9±24.9	--	--	886.3±40.5
	Girl:18.7±1.0	--	1.86±0.28	--	--	--	1.28±0.18	1.69±0.69	--	--	660.2±108.5	338.9±38.5	200.1±31.5	--	--	960±34.4
Chevaley 2017	22.6±0.8	0.186±0.003	2.14±0.32	0.088±0.012	0.390±0.065	--	1.37±0.3	4.09±1.24	--	--	--	346±51	223±33	--	--	887±26
Rudang 2013	18.9±0.6	0.185±0.027	2.09±0.26	0.089±0.011	0.397±0.059	--	1.332±0.30	3.03±1.18	0.168±0.02	--	--	--	--	--	--	874±33

BV/TV: trabecular bone volume to total volume fraction; Tb.N (1/mm): Trabecular number; Tb.Th: trabecular thickness (mm); Tb.Sp: trabecular separation (mm); Ct.Th: cortical thickness (mm); Ct.Po: cortical porosity (%), Ct.Ar: cortical area (mm^2^); Tt.Ar: total area (mm^2^); Ct.vBMD: cortical volumetric bone mineral density (mg/cm^3^); Tb.vBMD: trabecular volumetric bone mineral density (mg/cm^3^); and Tt.vBMD: total bone mineral density (mg/cm^3^).

Study characteristics included first author’s last name, year of publication, and questions. Patients’ demographic information included number, sex, mean height, mean weight, BMI and pubertal status. HR-pQCT information included the scanner brand, and references used in tibia and radius. The following HR-pQCT parameters automatically provided by HR-pQCT were collected: trabecular bone volume to total volume fraction (BV/TV); trabecular number (Tb.N); trabecular thickness (Tb.Th); trabecular separation (Tb.Sp); cortical thickness (Ct.Th); cortical porosity (Ct.Po), cortical area (Ct.Ar); total area; (Tt.Ar), cortical bone mineral density (Ct.vBMD); trabecular bone mineral density (Tb.vBMD) and total bone mineral density (Tt.vBMD).

### Quality assessment

Two readers (D.M.M and R.V.) who were unblinded to the journal names, author names, and year of publication assessed the reporting quality by using the Standards for Reporting of Diagnostic Accuracy (STARD) scoring systems [20]. To assess the methodology and risk of bias of included studies, Quality Assessment of Diagnostic Accuracy Studies 2 (QUADAS-2) was not used because none of these studies used a reference imaging test (micro computed tomography) or bony biopsy to compare with [21,22]. Instead, the Modified Newcastle-Ottawa scale (NOS) for case-control studies was used [23]. Each article was assessed independently by the two readers after a tutorial meeting on guidelines for the interpretation of items. Disagreements were resolved by consensus discussion with a third experienced reviewer (A.S.D.).

Scores from the STARD system were reported as a percentage of a maximum of 25 points [24]. S2, S3 and S4 Appendices contain STARD scoring systems and the scores of the articles. The 25 domains included in STARD were either assigned a score of 1 (adequately reported), 0.5 (partially reported) or 0 (not reported) for a maximum score of 25 [25,26]. Qualities that were not applicable were not assigned a numeric score and were marked as ‘n/a’ and their score was removed from the maximum score. For example, if one item was not applicable for a given study, the maximum STARD score was then 24. For detailed criteria for each item of STARD, please refer to S2 Appendix. Using the STARD tool, the reporting quality was determined based on the ratio of the overall score to the total applicable score for each assessment tool. Studies with ratios ≥90%, were classified as having high; <90% and ≥70%, moderate; <70% and ≥60%, low and <60%, very low reporting [26].

The NOS was evaluated based on the 3 main categories including the selection, comparability and exposure [23]. A study could be awarded a maximum of one star (letter A) for each numbered item within the Selection and Exposure categories. A maximum of two stars can be given for Comparability. For details about scoring NOS, please refer to S5, S6 and S7 Appendices. Finally, the overall NOS score was converted into the study quality following Agency for the healthcare research and quality (AHRQ) standards as follows published literature [27,28]: (a) Good quality: 3 or 4 stars (= letter A) in selection domain and 1 or 2 stars in comparability domain and 2 or 3 stars in outcome/exposure domain; (b) Fair quality: 2 stars in selection domain and 1 or 2 stars in comparability domain and 2 or 3 stars in outcome/exposure domain, and (d) Poor quality: 0 or 1 star in selection domain OR 0 stars in comparability domain OR 0 or 1 stars in outcome/exposure domain. The results of NOS scores of each article are shown in S7 Appendix.

### Analysis and statistics

Intraclass correlation coefficients were calculated for assessment of inter-reader agreement on STARD and NOS scores.

For the meta-analysis, we combined estimates and standard deviations of the 4 studies concerning data from males and females. Aggregated effect size using fixed effect and random effect methods were calculated. The inverse of the standard deviation was used for weighting. None of the Tau-squared was statistically significant, therefore we use fixed effect aggregated summary statistics and their 95% confidence intervals. Between-study heterogeneity was estimated using I^2^ statistic.

Statistical analysis was performed by using statistical software (SAS version 9.4; SAS Institute, Cary, NC). A P value less than .05 was used as the threshold to indicate statistical significance.

After considering the quality of the included studies, and heterogeneity between the included studies, levels of recommendation regarding the use of HR-PQCT in normal subjects were assigned according to the U.S. Preventive Services Task Force guidelines [29]. The guidelines are described in S8 and S9 Appendices.

## Results

### Literature search and article selection

Fig 1 shows the article selection process. The search yielded 3958 articles. After screening titles and abstracts, and removing duplications, the full text of 139 articles was reviewed. Eight articles [6,13,17–19, 30–32], with a total of 1308 patients, were ultimately selected for inclusion in the systematic review. Of the eight articles, only two studies included exclusively subjects aged less than 18 years [30,31], the remaining 6 studies [6, 13, 17–19, 32] included both subjects aged less 18 years and young adults (aged between 18 and 25 years). Of the eight studies, 6 included both male and females, while one included only females [16] and another one only males [13].

### Data extraction

Tables 1–3 contain basic study information, demographic data including maturity of patients and basic HR-pQCT parameters. All studies used the same HR-pQCT scanner (xtremeCT I, Scanco Medical, Switzerland), voxel size (82 μm3) and number of slices (110). Except one study which examined only radius [30], the remaining 7 studies evaluated both radius and tibia.

The Tables 2 and 3 show the details values of HR-pQCT parameters for each paper both at tibia and radius.

### Quality assessment of selected articles

Of the eight articles, 2 were judged of high [18,19], 5 of moderate [6,13,30–32] and 1 of [17] low reporting quality based on STARD. The STARD items 5 and 21 received the lowest scores (1/8 and 3/8, respectively). S2, S3 and S4 Appendices contain the results of the assessment of the methodologic and reporting quality of the studies by using the STARD scoring systems and contains detailed descriptions of the STARD scoring systems along with complete results from the quality assessment of each article.

Seven [6,13,17–19,30,32] out of 8 articles included in this systematic review were judged of good quality regarding their methodology and risk of bias based on NOS for case-controls. One article [31] did not fit the NOS for cohort or for case-controls. Results of NOS scores are shown in S7 Appendix. All the 4 papers [13, 17–19] included in subgroup meta-analysis were all judged of good quality based on NOS.

### Fixed- and random-effects models and I^2^ in meta-analysis

Four articles, encompassing a total of 713 patients, were ultimately combined in the meta-analysis part of this study. The details of results of Fixed and random-effects models of HR-pQCT parameters of the 4 articles based on subjects aged 15 to 22.6 years are shown in Tables 4 and 5.

**Table 4:** Aggregated effect size of HR-pQCT parameters of data of the 4 included papers using fixed and random effect methods: Radius.

Parameter	Fixed Effect Effect Size			Random Effect Summary				
	Estimate	Lower	upper	Estimate	Lower	Upper	T-squared	Radon P Value
BT/BV	0.1672	0.1229	0.2115	0.1672	0.1229	0.2115	2.1808E-8	0.49975
Tb_N	2.0807	2.0355	2.1259	2.0807	2.0355	2.1259	0	---
Tb.Th	0.07646	0.07082	0.08209	0.07693	0.07027	0.08359	.000004	0.31887
Tb.Sp	0.4073	0.3871	0.4275	0.4073	0.3871	0.4275	0	---
Ct.Th	0.8568	0.7644	0.9492	0.8568	0.7644	0.9492	0	---
Ct.Po	1.4653	0.5154	2.4151	1.5390	0.6373	2.4408	0.13177	0.15866
Ct.Ar	52.8412	---	---	---	---	---	---	---
Tt.Ar	255.34	-384.80	895.49	263.66	-385.31	912.63	5165.91	0.24192
Tt-VBMD	266.77	47.8913	485.64	280.46	73.1813	487.74	6962.58	0.15866
Tb-vBMD	223.60	47.1013	400.09	223.60	47.1013	400.09	0	---
CT.vBMD	765.97	389.07	1142.87	765.97	389.07	1142.87	0	---

Abbreviations: BV/TV: trabecular bone volume to total volume fraction; Tb.N (1/mm): Trabecular number; Tb.Th: trabecular thickness (mm); Tb.Sp: trabecular separation (mm); Ct.Th: cortical thickness (mm); Ct.Po: cortical porosity (%), Ct.Ar: cortical area (mm^2^); Tt.Ar: total area (mm^2^); Ct.vBMD: cortical volumetric bone mineral density (mg/cm^3^); Tb.vBMD: trabecular volumetric bone mineral density (mg/cm^3^); and Tt.vBMD: total bone mineral density (mg/cm^3^).

**Table 5:** Aggregated effect size of HR-pQCT parameters of data of the 4 included papers using fixed and random effect methods: Tibia.

Parameter	Fixed Effect Effect Size			Random Effect Summary				
	Estimate	Lower	upper	Estimate	Lower	Upper	T-squared	Radon P Value
BT/BV	0.1859	0.1821	0.1897	0.1855	0.1791	0.1919	.0000005	---
Tb_N	2.0215	1.8366	2.2064	2.0215	1.8366	2.2064	0	---
Tb.Th	0.08892	0.08654	0.09130	0.08892	0.08654	0.09130	0	---
Tb.Sp	0.4035	0.3645	0.4424	0.4021	0.3640	0.4403	.0002360	0.15866
Ct.Th	1.3216	1.2649	1.3782	1.3253	1.2684	1.3823	.001279	0.11109
Ct.Po	3.1580	1.1290	5.1870	3.1580	1.1290	5.1870	0	---
Ct.Ar	125.65	---	---	---	---	---	---	---
Tt.Ar	689.81	148.72	1230.90	693.08	150.29	1235.87	3531.89	0.24691
Tt-VBMD	343.58	335.06	352.11	343.76	335.45	352.08	11.2128	0.15866
Tb-vBMD	213.37	193.47	233.27	213.37	193.47	233.27	0	---
CT.vBMD	894.16	855.01	933.32	894.32	857.57	931.06	533.303	0.11034

Abbreviations: BV/TV: trabecular bone volume to total volume fraction; Tb.N (1/mm): Trabecular number; Tb.Th: trabecular thickness (mm); Tb.Sp: trabecular separation (mm); Ct.Th: cortical thickness (mm); Ct.Po: cortical porosity (%), Ct.Ar: cortical area (mm^2^); Tt.Ar: total area (mm^2^); Ct.vBMD: cortical volumetric bone mineral density (mg/cm^3^); Tb.vBMD: trabecular volumetric bone mineral density (mg/cm^3^); and Tt.vBMD: total bone mineral density (mg/cm^3^).

The estimate of HR-pQCT paramaters using random-effect models were as follows: For the radius: BT/BV estimate was 0.17: 0.1229(lower)-0.2115 (upper); Tb_N: 2.08 (2.03–2.12) 1/mm; Tb.Th: 0.07 (0.07–0.0.08) mm; Tb.Sp: 0.41 (0.38–0.42) mm; Ct.Th: 0.85 (0.76–0.94) mm; Ct.Po: 1.53 (0.63–2.44)%; Tt.Ar: 263.66(-385.3–912.6) mm^2^; Tt-vBMD: 280.5 (73.1–487.7) mg/cm^3^; Tb-vBMD: 223.6 (47.1–400.09) mg/cm^3^ and CT.vBMD: 765.9 (389.1–1142.8) mg/cm^3^. For the Tibia: BT/BV: 0.18 (0.17–0.19); Tb_N: 2.02 (1.83–2.2) 1/mm; Tb.Th: 0.08 (0.80–0.09) mm; Tb.Sp: 0.40(0.36–0.44) mm; Ct.Th: 1.32(1.26–1.38) mm; Ct.Po: 3.15 (1.1–5.2)%; Tt.Ar: 693.1 (150.2–1235.8) mm^2^; Tt-vBMD: 343.76 (335.5–352.1) mg/cm^3^;Tb-vBMD: 213.37 (193.5–233.2) mg/cm^3^ and CT.vBMD: 894.3 (857.6–931.1) mg/cm^3^.

The I2 for the outcomes are: 1) for the radius: BT/BV: 0, Tb_N:0; Tb.Th: 0; Tb.Sp: 0; Ct.Th: 0; Ct.Po: 0; Ct_ar: -one study only; Tt.Ar:0.99; Tt-vBMD: 0.99; Tb-vBMD: 0.99 and CT.vBMD: 1.0. For the Tibia: BT/BV: 0, Tb_N:0; Tb.Th: 0; Tb.Sp: 0; Ct.Th: 0; Ct.Po: 0; Ct_ar: -one study only; Tt.Ar: 0.97; Tt-vBMD: 0; Tb-vBMD: 0.52 and CT.vBMD: 0.93

## Discussion

This systematic review and subgroup meta-analysis of HR-pQCT normative data in a pediatric, adolescent and young adult population, included 8 articles that were selected for rating based on a priori determined inclusion and exclusion criteria.

Subgroup meta-analysis of four articles that included adolescents and young adults aged 15 to 22.6 years (corresponding to 713 patients) using a random effects model, yielded estimates for normative data in this subgroup population for HR-pQCT parameters including: bone volume fraction, trabecular number, trabecular thickness, trabecular separation, cortical thickness, cortical porosity, total area, total bone density, trabecular density and cortical density (Tables 4 and 5). These results were generated for both the distal radius and tibia. We concluded that there was a fair recommendation, based on the U.S. Preventive Service Task Force, for clinicians to routinely recommend the performance of HR-pQCT to eligible patients, based on evidence of aggregate HR-pQCT parameters from a healthy population aged 15 to 22.6 years (from cohort or controls in case-control studies), as well as on associations reported between HR-pQCT values and clinical parameters such as sex, body mass index, and serum sclerostin levels [17,32].

To our knowledge, this study is the first to aggregate, summarize and analyze the existing literature surrounding HR-pQCT values in a healthy pediatric and young adult population. Such a study is crucial for assessing the full potential of HR-pQCT scanning in a clinical setting, so that there may be normal comparisons for HR-pQCT parameters for a young population in order to reliably and accurately identify pathologies or indicators of poor bone quality. Having established standards for comparison in specific age groups of adolescent and young adults (aged 15 to 22.6 years) is particularly important, as bone parameters vary greatly over the childhood, adolescent and young adult period, especially in comparison to adulthood, due to pubertal status and fluctuating hormone levels [14,33,34]. To this end, we believe that the results of our meta-analysis are of paramount clinical importance because the bony parameters of subjects between the ages 15 to 22.6 years vary less, knowing that at around 15 years, individuals are skeletally mature, similar to those of adults. This could serve of as reference in clinical use and will direct future studies in younger patients, as our data demonstrate that there is scarce normative data for HR-pQCT parameters, especially in children. There is still much we do not know with regards to the utility of HR-pQCT in the growing skeleton, especially regarding the relationship between bone structure and strength in childhood and propensity to disease such as fractures later in life [35,36]. Thus, this requires urgent standardization of acquisition parameters and guidelines on the use of HR-pQCT in these populations.

One of the strengths of this study is that included articles used similar techniques. Of note, nowadays, there are two different generations of HR-pQCT scanners used in practice including xtremeCT-1 and xtreme CT-2 scanners [37]. However, only the xtremeCT-1 was used in all 8 included articles. In addition, with regards to HR-pQCT data acquisition, there are mainly two protocols regarding how to select the regions of interest. Most of the included studies use either a fixed distance from the end/growth plate or percentage of distance of bone length of the non-dominant radius or tibia. Of the 8 articles included, only one [30] article used the percentage of the distance of bone length. The remaining 7 articles used the distance from the end of the bone. Specifically, the 4 articles summarized in the meta-analyses used the same references, that is, the first computed tomography slice at the distal radius and tibia was 9 and 22.5 mm proximal to the reference line, respectively. We believe that the results of our meta-analyses are robust because they are based on articles with minimal technical variations, which ensures that our findings are generalizable.

Limitations of the current study include the relatively small quantity of studies published that included HR-pQCT parameters for a healthy pediatric and young adult population. Also, few studies were dedicated to HR-pQCT results in a specifically pediatric (18 years of age or younger) population, rendering it difficult to analyze reference values for this age cohort separately. Moreover, the overall age range in the systematic review part of this paper is broad, and presumably norms will vary tremendously for a 1-year-old versus a 25-year-old, which could limit the usefulness of our findings. However, we believe that this paper has merit in providing at least the normal range of HR-pQCT parameters in adolescent and young adults (15–22.6 years of age), in which there is less variation in bony structures, knowing that at around 15 years of age individuals are skeletally mature. This could guide future efforts to establish reference values in younger patients. The heterogeneous nature of aggregating results from multiple studies that lack widely adopted standardized references and protocols for HR-pQCT scanning, and the fact that none of included studies provided information regarding operators’ training and scanner cross calibration also served to limit the current study. In addition, studies included in this meta-analysis used a fixed-offset scan position rather than a percentage offset position knowing that the two techniques will yield different outcome parameter values and the percentage offset method could possibly be the most appropriate and/or the most common technique in scanning children. However, based on our pre-set inclusion criteria, only studies with the fixed-offset methodology were included. Since both methods are still used, our result could serve the reference for all centers at this time. Further studies summarizing data from percentage offset position are advocated. This meta-analysis was not registered online, which also served to limit the current study. Due to these limitations, future work should focus on generating standardized HR-pQCT protocols for various bone regions. Additionally, further studies are required to document normative HR-pQCT parameters in a pediatric population in order to further validate established reference values that may be age-matched for patients undergoing HR-pQCT scanning.

In conclusion, there is overall fair evidence for our reported results for normative data of HR-pQCT parameters in children, adolescents and/or young adults. Our study illustrates the scarcity of available data in the literature, and emphasize the need for standardization of acquisition parameters and guidelines on the use of HR-pQCT in these populations.

## Supporting information

S1 Appendix Search strategy for identification of studies that satisfied the inclusion criteria of this meta-analysis. (DOCX)

S2 Appendix Detailed criteria on the Standard for Reporting of Diagnostic Accuracy (STARD) assessment. (DOCX)

S3 Appendix Assessment of quality of reporting, methodological quality and categorization of primary studies. (DOCX)

S4 Appendix Assessment of Standard for Reporting of Diagnostic Accuracy (STARD) items of included studies. (DOCX)

S5 Appendix Newcastle-Ottawa Quality assessment scale. (DOCX)

S6 Appendix Coding Manual for Case-Control Studies. (DOCX)

S7 Appendix Newcastle—Ottawa Quality Assessment Scale Case-Control Studies. (DOCX)

S8 Appendix Levels of recommendation of results according to the guidelines of the U.S. Preventive Service Task Force. (DOCX)

S9 Appendix Categorization of study design according to the U.S. Preventive Service Task Force (USPSTF). (DOCX)

## Abbreviations

HR-pQCT: High resolution peripheral quantitative computed tomography
DXA: Dual Energy X-ray Absorptiometry
BMD: bone mineral density
BV/TV: trabecular bone volume to total volume fraction
Tb.N: Trabecular number
Tb.Th: trabecular thickness
Tb.Sp: trabecular separation
Ct.Th: cortical thickness
Ct.Po: cortical porosity
Ct.Ar: cortical area
Tt.Ar: total area
Ct.vBMD: cortical volumetric bone mineral density
Tb.vBMD: trabecular volumetric bone mineral density
Tt.vBMD: total bone mineral density
