# Supplementary material for: HR-pQCT imaging in children, adolescents and young adults: Systematic review and subgroup meta-analysis of normative data
Source: PLoS One. 2019 Dec 13;14(12):e0225663. doi: 10.1371/journal.pone.0225663 (PMC6910691; doi:10.1371/journal.pone.0225663)
Supplement: S1 Appendix — (DOCX) [file pone.0225663.s001.docx]

**Supplementary materials**

**S1 Appendix: Search strategy for identification of studies that satisfied the inclusion criteria of this meta-analysis.**

Searches were conducted in Ovid MEDLINE Epub Ahead of Print, In-Process & Other Non-Indexed Citations, Ovid MEDLINE Daily, and Ovid MEDLINE (1946 to July 2019) and Embase Classic + Embase < 1947 to 2019 Week 30.

**S1 Table: Summary data of patients of any age.**

| **Databases [Platform]** | **Results** |
| --- | --- |
| MEDLINE(R) Epub Ahead of Print, In-Process & Other Non-Indexed Citations, MEDLINE(R) Daily and MEDLINE(R) 1946 to Present [Ovid] | 642+322=964 |
| **Embase Classic+Embase**1947 to 2017 Week 41 [Ovid] | 1347+544=1891 |
| **Cochrane Database of Systematic Reviews**2005 to October 5, 2017 [Ovid] | 2+0=2 |
| [Web of Science]  Science Citation Index Expanded (SCI-EXPANDED) --1900-present  Social Sciences Citation Index (SSCI) --1956-present  Conference Proceedings Citation Index- Science (CPCI-S) --1990-present  Conference Proceedings Citation Index- Social Science & Humanities (CPCI-SSH) --1990-present  Emerging Sources Citation Index (ESCI) --2015-present | 777+324=1101 |
| **TOTAL** | **2768+1190=3958** |

**S2 Table: Summary data of patients under 18 years of age**

| **Databases [Platform]** | **Results** |
| --- | --- |
| MEDLINE(R) Epub Ahead of Print, In-Process & Other Non-Indexed Citations, MEDLINE(R) Daily and MEDLINE(R) 1946 to Present [Ovid] | 99+66=165 |
| **Embase Classic+Embase**1947 to 2017 Week 41 [Ovid] | 186+91=277 |
| **Cochrane Database of Systematic Reviews**2005 to October 5, 2017 [Ovid] | 2+0=2 |
| [Web of Science]  Science Citation Index Expanded (SCI-EXPANDED) --1900-present  Social Sciences Citation Index (SSCI) --1956-present  Conference Proceedings Citation Index- Science (CPCI-S) --1990-present  Conference Proceedings Citation Index- Social Science & Humanities (CPCI-SSH) --1990-present  Emerging Sources Citation Index (ESCI) --2015-present | 98+56=154 |
| **TOTAL** | **385+213=598** |

**S3 Table: MEDLINE(R) Epub Ahead of Print, In-Process & Other Non-Indexed Citations, MEDLINE(R) Daily and MEDLINE(R)**1946 to Present 

Search Strategy:

| **#** | **Searches** | **Results** |
| --- | --- | --- |
| 1 | ((((high adj2 resolut*) or highresolution or "hr") adj2 peripheral adj2 quantitative adj2 computed adj2 tomograph*) or "hrpqct" or "hr pqct").mp. | 812 |
| 2 | ("high resolution pqct" or "highresolution pqct" or "hr-pqct" or "hrpqct").mp. | 704 |
| 3 | 1 or 2 | 836 |
| 4 | (infan* or newborn* or "new born*" or perinat* or neonat* or baby or baby* or babies or toddler* or minors* or boy or boys or boyfriend or boyhood or girl* or kid or kids or child* or schoolchild* or adolescen* or juvenil* or youth* or teen* or "under* age*" or pubescen* or pediatric* or paediatric* or peadiatric* or prematur* or preterm*).mp. or pediatrics/ | 4312878 |
| 5 | 3 and 4 | 139 |
| 6 | ("20171005" or "20171006" or "20171007" or "20171008" or "20171009" or 2017101* or 2017102* or 2017103* or 201711* or 201712* or 2018* or 2019*).dt,ez,ed. | 3215621 |
| 7 | 5 and 6 | 66 |
| 8 | remove duplicates from 7 | 66 |
| 9 | 3 and 6 | 322 |
| 10 | remove duplicates from 9 | 322 |

**S4 Table: Embase Classic+Embase 1947 to 2019 Week 30**

Search Strategy:

| **#** | **Searches** | **Results** |
| --- | --- | --- |
| 1 | ((((high adj2 resolut*) or highresolution or "hr") adj2 peripheral adj2 quantitative adj2 computed adj2 tomograph*) or "hrpqct" or "hr pqct").mp. | 1901 |
| 2 | ("high resolution pqct" or "highresolution pqct" or "hr-pqct" or "hrpqct").mp. | 1770 |
| 3 | 1 or 2 | 1957 |
| 4 | (infan* or newborn* or "new born*" or perinat* or neonat* or baby or baby* or babies or toddler* or minors* or boy or boys or boyfriend or boyhood or girl* or kid or kids or child* or schoolchild* or adolescen* or juvenil* or youth* or teen* or "under* age*" or pubescen* or pediatric* or paediatric* or peadiatric* or prematur* or preterm*).mp. or pediatrics/ | 4855522 |
| 5 | 3 and 4 | 276 |
| 6 | limit 5 to dc=20171005-20190729 | 91 |
| 7 | remove duplicates from 6 | 91 |
| 8 | limit 3 to dc=20171005-20190729 | 549 |
| 9 | remove duplicates from 7 | 544 |

**S5 Table: Cochrane Database of Systematic Reviews 2005 to 2019 Week 30** 
Search Strategy:

| **#** | **Searches** | **Results** |
| --- | --- | --- |
| 1 | ((((high adj2 resolut*) or highresolution or "hr") adj2 peripheral adj2 quantitative adj2 computed adj2 tomograph*) or "hrpqct" or "hr pqct").mp. | 2 |
| 2 | ("high resolution pqct" or "highresolution pqct" or "hr-pqct" or "hrpqct").mp. | 2 |
| 3 | 1 or 2 | 2 |
| 4 | (infan* or newborn* or "new born*" or perinat* or neonat* or baby or baby* or babies or toddler* or minors* or boy or boys or boyfriend or boyhood or girl* or kid or kids or child* or schoolchild* or adolescen* or juvenil* or youth* or teen* or "under* age*" or pubescen* or pediatric* or paediatric* or peadiatric* or prematur* or preterm*).mp. or pediatrics/ | 6317 |
| 5 | 3 and 4 | 2 |
| 6 | (201711* or 2018* or 2019*).up. | 2491 |
| 7 | 5 and 6 | 0 |

**S6 Table: Web of Science**

**Web of Science**

Science Citation Index Expanded (SCI-EXPANDED) --1900-present

Social Sciences Citation Index (SSCI) --1956-present

Conference Proceedings Citation Index- Science (CPCI-S) --1990-present

Conference Proceedings Citation Index- Social Science & Humanities (CPCI-SSH) --1990-present

Emerging Sources Citation Index (ESCI) --2015-present

| **Set** | **Results** | **Save History / Create AlertOpen Saved History** |
| --- | --- | --- |
| # 6 | **324** | #3 NOT #5  *Indexes=SCI-EXPANDED, SSCI, CPCI-S, CPCI-SSH, ESCI Timespan=2017-2019* |
| # 5 | **56** | #4 AND #3  *Indexes=SCI-EXPANDED, SSCI, CPCI-S, CPCI-SSH, ESCI Timespan=2017-2019* |
| # 4 | **3142055** | TS=(infan* or newborn* or "new born*" or neonat* or baby* or babies or toddler* or minors* or boy or boys or boyfriend or boyhood or girl* or kid or kids or child* or adolescen* or juvenil* or youth* or teen* or "under* age*" or pubescen* or pediatric* or paediatric* or peadiatric* or prematur* or preterm*)  *Indexes=SCI-EXPANDED, SSCI, CPCI-S, CPCI-SSH, ESCI Timespan=1900-2019* |
| # 3 | **1169** | #2 OR #1  *Indexes=SCI-EXPANDED, SSCI, CPCI-S, CPCI-SSH, ESCI Timespan=1900-2019* |
| # 2 | **1024** | TS=("high resolution pqct" or "highresolution pqct" or "hr-pqct" or "hrpqct")  *Indexes=SCI-EXPANDED, SSCI, CPCI-S, CPCI-SSH, ESCI Timespan=1900-2019* |
| # 1 | **1131** | TS=((((high NEAR/2 resolut*) or highresolution or "hr") NEAR/2 peripheral NEAR/2 quantitative NEAR/2 computed NEAR/2 tomograph*) or "hrpqct" or "hr pqct")  *Indexes=SCI-EXPANDED, SSCI, CPCI-S, CPCI-SSH, ESCI Timespan=1900-2019* |
