# Supplementary material for: HR-pQCT imaging in children, adolescents and young adults: Systematic review and subgroup meta-analysis of normative data
Source: PLoS One. 2019 Dec 13;14(12):e0225663. doi: 10.1371/journal.pone.0225663 (PMC6910691; doi:10.1371/journal.pone.0225663)
Supplement: S2 Appendix — (DOCX) [file pone.0225663.s002.docx]

**S2 Appendix**: **Detailed criteria on the Standard for Reporting of Diagnostic Accuracy (STARD) assessment (i, ii).**

| **Item #** | **Item1** | **Item2** | **Item3** | **Item4** | **Item5** |
| --- | --- | --- | --- | --- | --- |
| **Description** | Identify the article as a study of diagnostic accuracy | State the research questions or study aims (in abstract or introduction) | Inclusion/exclusion criteria + location | Describe participant recruitment | Participant sampling: consecutive series defined by selection criteria in (3) and (4)? if not, state how it was done (in method & material) |
| **Score 1** | See: ROC, predictive value, true/false positive, true/false negative, Sensitivity, Specificity, correlation, reliability, responsiveness. | The research question helps the reader to predict the method used (including the tools and statistics they will be using). ("compare" is good enough) | Must have at least one inclusion/exclusion criteria, AND location of study. | Recruitment baised on presenting symptoms or results from previous tests | consecutive/random/all patient within a period of time |
| **Score 0.5** | -- | -- | One of inclusion/exclusion criteria, OR location of study | -- |  |
| **Score 0** | No key word found | The research question misleads reader or leaves unanswered questions. | Missing inclusion/exclusion criteria AND location. | When readers cannot answer the above question | Not reported |
| **N/A** | -- | -- | -- | -- | -- |

| **Item #** | **Item6** | **Item7** | **Item8** | **Item9** | **Item10** |
| --- | --- | --- | --- | --- | --- |
| **Description** | Data collection planned before the index test (prospective) or after (retrospective) | What is the reference standard and what's the rationale | Describe the tests and reference standard | Definition and rationale for units and cutoffs (for the reference standard) | Number and training of operators for both the index test and reference standard |
| **Score 1** | If the readers understand whether it is a prospective or retrospective study (implicit indication is fine) | state what is the reference standard and the rational | Details that enable reader to reproduce all tests, except for information about operators | Parameter, unit, cutoff, and their rationales when necessary | Both number and training for all scans |
| **Score 0.5** | -- | -- | -- | -- | Only one of number or training |
| **Score 0** | When readers cannot understand whether it is a prospective or retrospective study | Not reported | When there is not enough details for readers to reproduce the scans |  | Missing both |
| **N/A** | -- | When the research question does not include criterion validity. | -- | Same as item 7 | -- |

| **Item #** | **Item11** | **Item12** | **Item13** | **Item14** | **Item15** |
| --- | --- | --- | --- | --- | --- |
| **Description** | Who and what was blinded? | Describe the statistics used | Method of calculating reliability | Beginning and ending dates of recruitment | Demographic characteristics |
| **Score 1** | Indicate whether the operators and readers are blinded | Exactly what statistical tests are used | If we can reproduce the design (name of statistics, and how data was collected for this purpose) Note: vague names like “precision” are not acceptable | Both dates | Age, gender |
| **Score 0.5** |  | -- | Missing one of the name of the statistics used, or the data collection | -- | Age or gender (1 item only) |
| **Score 0** | No indication of whether the operators and readers are blinded | No explanation of statistical methods | Missing both | Missing either date | Not Reported |
| **N/A** | -- | -- | -- | -- | -- |

| **Item #** | **Item16** | **Item17** | **Item18** | **Item19** | **Item20** |
| --- | --- | --- | --- | --- | --- |
| **Description** | Describe why some participants failed to receive the test | Time interval and any tx administered between index and reference standard | distribution of severity of disease in those with target condition (osteoporosis, BMD values) - valid for physical activity | Cross tabulation of index test results by the results of the reference standard | Adverse events (e.g. radiotion) N/a = when not in the context of the research question |
| **Score 1** | Explain why some patients didn't get scan, or explicit statement that all recruited patients were successfully scanned | Time interval between all testes is reported | Spectrum of (primary) disease severity OR subtypes of the disease. Subgrouping is not necessary | Enough to reproduce the statistics for diagnostic accuracy | -- |
| **Score 0.5** |  | -- | -- | -- | -- |
| **Score 0** | Not reported | Time interval between all testes is not reported | Not enough details to score 1. | Not enough to reproduce the statistics for diagnostic accuracy | -- |
| **N/A** | -- | Same as item 7 | -- | Same as item 7 | -- |

| **Item #** | **Item21** | **Item22** | **Item23** | **Item24** | **Item25** |
| --- | --- | --- | --- | --- | --- |
| **Description** | Uncertainty of result | How indeterminate results, missing data, and outliers of the index tests were handled. | Report difference of results in different subgroups of participants | The test result of reproducibility, which statisitcs? (CV value, ICC value) | Discuss clinical applications |
| **Score 1** | Report the uncertainty of results pertaining to the main objective(CI) | If the process was reported | Report difference of result (pertaining to the main objective) in different subgroups | There's reporting of the result of reliability (specify CV or ICC) | 1. Report the limitation of the study 2. Interpretation of result in clinical setting |
| **Score 0.5** | -- | -- | -- | -- | Reported one of the two |
| **Score 0** | No uncertainty reported | If not reported | Either results of different subgroups were not reported, or there was no subgrouping of participant | There was no reporting of the result of reliability | Neither was reported |
| **N/A** | -- | -- | -- | -- | -- |
