# Supplementary material for: HR-pQCT imaging in children, adolescents and young adults: Systematic review and subgroup meta-analysis of normative data
Source: PLoS One. 2019 Dec 13;14(12):e0225663. doi: 10.1371/journal.pone.0225663 (PMC6910691; doi:10.1371/journal.pone.0225663)
Supplement: S3 Appendix — (DOCX) [file pone.0225663.s003.docx]

**S3 Appendix: Assessment of quality of reporting, methodological quality and categorization of primary studies.**

| **First author’s last name/ publication year** | **Children/adult** | **Theme** | **STARD** | | | | | | | **USPSTF** |
| --- | --- | --- | --- | --- | --- | --- | --- | --- | --- | --- |
|  |  |  | **Reporting quality** | **Percentage score** | **Raw score** | **Not reported** | **Partially reported** | **Reported** | **Items evaluated**  **(max 25)** |  |
| Cheuk 2016 | pediatric | Comparison between two HR-pQCT protocols | Moderate | 73.30% | 11 | 4 | 0 | 11 | 15 | II-2 |
| [Ackerman](https://www.ncbi.nlm.nih.gov/pubmed/?term=Ackerman%20KE%5BAuthor%5D&cauthor=true&cauthor_uid=21816790) 2011 | pediatric | Bone microarchitecture in amenorrheic athletes compared to  eumenorrheic athletes (EA) and nonathletic controls. | low | 66.70% | 10 | 5 | 0 | 10 | 15 | II-2 |
| Kawalilak 2017 | pediatric | Definition of HR-pQCT precision errors and least significant changes at the distal radius and tibia in children | Moderate | 73.30% | 11 | 4 | 0 | 11 | 15 | II-2 |
| Gabel 2017 | Pediatric and young adult | Describe and compared between boy and girl growth related adaptations in bone microarchitecture, geometry, density, and strength at the distal tibia and Radius. | Moderate | 86.70% | 13 | 2 | 0 | 13 | 15 | II-2 |
| Kirmani 2012 | Pediatric and young adult | Changes in circulating sclerostin and correlation sclerostin levels with bone microarchitecture, bone turnover markers, and serum hormone levels during pubertal growth. | Moderate | 73.30% | 11 | 4 | 0 | 11 | 15 | II-2 |
| Burt  2014 | Pediatric and young adult | To determine if bone microstructural and strength parameters identified by HR-pQCT and finite element analysis at the distal radius and tibia, peak within the age range of this youth cohort, and whether the timing of the peaks differ based on sex or skeletal site. | High | 93.70% | 15 | 1 | 0 | 15 | 16 | II-2 |
| Chevaley  2017 | Young adults | To assess in healthy males the relationship between fracture during development and expression of bone fragility in adulthood. | Moderate | 85.70% | 12 | 2 | 0 | 12 | 14 | II-2 |
| Rudang  2013 | Young adults | To investigate whether prevalent fractures, occurring from birth to young adulthood, were related to HR‐pQCT, derived trabecular and cortical microstructure, and bone strength estimated by finite element. | High | 92.90% | 13 | 1 | 0 | 13 | 14 | II-2 |

HR-pQCT: High resolution peripheral quantitative computed tomography
