# Supplementary material for: HR-pQCT imaging in children, adolescents and young adults: Systematic review and subgroup meta-analysis of normative data
Source: PLoS One. 2019 Dec 13;14(12):e0225663. doi: 10.1371/journal.pone.0225663 (PMC6910691; doi:10.1371/journal.pone.0225663)
Supplement: S4 Appendix — (DOCX) [file pone.0225663.s004.docx]

**S4 Appendix: Assessment of Standard for Reporting of Diagnostic Accuracy (STARD) items of included studies.**

| **First author’s last name/ publication year** | **STARD Item Number** | | | | | | | | | | | | | | | | | | | | | | | | | **Total** | **%** |
| --- | --- | --- | --- | --- | --- | --- | --- | --- | --- | --- | --- | --- | --- | --- | --- | --- | --- | --- | --- | --- | --- | --- | --- | --- | --- | --- | --- |
|  | 1 | 2 | 3 | 4 | 5 | 6 | 7 | 8 | 9 | 10 | 11 | 12 | 13 | 14 | 15 | 16 | 17 | 18 | 19 | 20 | 21 | 22 | 23 | 24 | 25 |  |  |
| Cheuk 2016 | 1 | 1 | 1 | 1 | 0 | 1 | n/a | 1 | n/a | n/a | n/a | 1 | 0 | n/a | 1 | 0 | n/a | n/a | n/a | n/a | 0 | 1 | 1 | n/a | 1 | 11 | 73.30 |
| [Ackerman](https://www.ncbi.nlm.nih.gov/pubmed/?term=Ackerman%20KE%5BAuthor%5D&cauthor=true&cauthor_uid=21816790) 2011 | 1 | 1 | 1 | 1 | 0 | 1 | n/a | 1 | n/a | n/a | n/a | 1 | n/a | 0 | 1 | 0 | n/a | n/a | n/a | n/a | 0 | 0 | 1 | n/a | 1 | 10 | 66.7 |
| Kawalilak 2017 | 1 | 1 | 0 | 0 | 0 | 1 | n/a | 1 | n/a | n/a | n/a | 1 | n/a | 0 | 1 | 1 | n/a | n/a | n/a | n/a | 1 | 1 | 1 | n/a | 1 | 11 | 73.3 |
| Gabel 2017 | 1 | 1 | 1 | 1 | 0 | 1 | n/a | 1 | n/a | n/a | n/a | 1 | n/a | 1 | 1 | 1 | n/a | n/a | n/a | n/a | 0 | 1 | 1 | n/a | 1 | 13 | 86.7 |
| Kirmani 2012 | 1 | 1 | 1 | 1 | 0 | 0 | n/a | 1 | n/a | n/a | n/a | 1 | n/a | 0 | 1 | 1 | n/a | n/a | n/a | n/a | 0 | 1 | 1 | n/a | 1 | 11 | 73.3 |
| Burt 2014 | 1 | 1 | 1 | 1 | 1 | 1 | n/a | 1 | n/a | n/a | n/a | 1 | n/a | 0 | 1 | 1 | 1 | n/a | n/a | n/a | 1 | 1 | 1 | n/a | 1 | 15 | 93.7 |
| Chevaley 2017 | 1 | 1 | 1 | 1 | 0 | 1 | n/a | 1 | n/a | n/a | n/a | 1 | n/a | 1 | 1 | 1 | n/a | n/a | n/a | n/a | 0 | 1 | n/a | n/a | 1 | 12 | 85.7 |
| Rudang 2013 | 1 | 1 | 1 | 1 | 0 | 1 | n/a | 1 | n/a | n/a | n/a | 1 | n/a | 1 | 1 | 1 | n/a | n/a | n/a | n/a | 1 | 1 | n/a | n/a | 1 | 13 | 92.9 |
| Total | 8/8 | 8/8 | 7/8 | 7/8 | 1/8 | 7/8 | n/a | 8/8 | n/a | n/a | n/a | 8/8 | 0/1 | 3/7 | 8/8 | 6/8 | 1/1 | n/a | n/a | n/a | 3/8 | 7/8 | 6/6 | n/a | 8/8 |  |  |
| Agreement DMM & RV | 100 | 100 | 100 | 100 | 100 | 100 | 100 | 100 | 100 | 100 | 100 | 100 | 100 | 100 | 100 | 100 | 100 | 100 | 100 | 100 | 100 | 100 | 100 | 100 | 100 |  |  |
