# Supplementary material for: HR-pQCT imaging in children, adolescents and young adults: Systematic review and subgroup meta-analysis of normative data
Source: PLoS One. 2019 Dec 13;14(12):e0225663. doi: 10.1371/journal.pone.0225663 (PMC6910691; doi:10.1371/journal.pone.0225663)
Supplement: S5 Appendix — (DOCX) [file pone.0225663.s005.docx]

**S5 Appendix: Newcastle-Ottawa Quality Assessment Scale case control studies**

Note: A study can be awarded a maximum of one star for each numbered item within the Selection and Exposure categories. A maximum of two stars can be given for Comparability.

**Selection**

1) Is the case definition adequate?

a) yes, with independent validation *****

b) yes, eg record linkage or based on self reports

c) no description

2) Representativeness of the cases

a) consecutive or obviously representative series of cases *****

b) potential for selection biases or not stated

3) Selection of Controls

a) community controls *****

b) hospital controls

c) no description

4) Definition of Controls

a) no history of disease (endpoint)

b) no description of source

**Comparability**

1) Comparability of cases and controls on the basis of the design or analysis

a) study controls for _______________ (Select the most important factor.) *****

b) study controls for any additional factor **** (This criteria could be modified to indicate specific control for a second important factor.)

**Exposure**

1) Ascertainment of exposure

a) secure record (eg surgical records) *****

b) structured interview where blind to case/control status *****

c) interview not blinded to case/control status

d) written self report or medical record only

e) no description

2) Same method of ascertainment for cases and controls

a) yes *****

b) no

3) Non-Response rate

a) same rate for both groups *****

b) non respondents described

c) rate different and no designation
