# Supplementary material for: HR-pQCT imaging in children, adolescents and young adults: Systematic review and subgroup meta-analysis of normative data
Source: PLoS One. 2019 Dec 13;14(12):e0225663. doi: 10.1371/journal.pone.0225663 (PMC6910691; doi:10.1371/journal.pone.0225663)
Supplement: S7 Appendix — (DOCX) [file pone.0225663.s007.docx]

**S7 Appendix: Newcastle - Ottawa Quality Assessment Scale Case-Control Studies**

| **First author’s last name/ publication year** | **Selection** | | | | **Comparability** | **Outcome** | | | |
| --- | --- | --- | --- | --- | --- | --- | --- | --- | --- |
|  | Is the case definition adequate? | Representativeness of the cases | Selection of Controls | Definition of Controls |  | Ascertainment of exposure | Same method of ascertainment for cases and controls | Non-Response rate | Agency for Healthcare Research and Quality |
| Cheuk 2016 | A | A | B | A | A | A | A | A | Good |
| [Ackerman](https://www.ncbi.nlm.nih.gov/pubmed/?term=Ackerman%20KE%5BAuthor%5D&cauthor=true&cauthor_uid=21816790) 2011***** | A | A | A | A | A | A | A | A | Good |
| Kawalilak 2017# | N/A | N/A | N/A | N/A | N/A | N/A | N/A | N/A | N/A |
| Gabel 2017 | A | A | A | A | A | A | A | A | Good |
| Kirmani 2012 | C | A | A | A | A | A | A | A | Good |
| Burt 2014***** | A | A | A | A | A | A | A | A | Good |
| Chevaley 2017***** | A | A | A | A | A | A | A | A | Good |
| Rudang 2013***** | A | A | A | A | A | A | A | A | Good |
| Agreement DMM & RV | 100% | 100% | 100% | 100% | 100% | 100% | 100% | 100% |  |

*Articles included in meta-analyses.

# Article not fitting the Ottawa Quality Assessment Scale Case-Control Studies or for cohort
