# Supplementary material for: HR-pQCT imaging in children, adolescents and young adults: Systematic review and subgroup meta-analysis of normative data
Source: PLoS One. 2019 Dec 13;14(12):e0225663. doi: 10.1371/journal.pone.0225663 (PMC6910691; doi:10.1371/journal.pone.0225663)
Supplement: S8 Appendix — (DOCX) [file pone.0225663.s008.docx]

**S8 Appendix: Levels of recommendation of results according to the guidelines of the U.S. Preventive Service Task Force.**

| 1. Strong recommendation for clinicians to routinely provide the service to eligible patients. 2. Fair recommendation for clinicians to routinely provide the service to eligible patients 3. No recommendation for or against routine provision of the service due to conflicting evidence 4. Recommendation against the provision of the service   I: Insufficient evidence to make recommendation |
| --- |
