# Supplementary material for: HR-pQCT imaging in children, adolescents and young adults: Systematic review and subgroup meta-analysis of normative data
Source: PLoS One. 2019 Dec 13;14(12):e0225663. doi: 10.1371/journal.pone.0225663 (PMC6910691; doi:10.1371/journal.pone.0225663)
Supplement: S9 Appendix — (DOCX) [file pone.0225663.s009.docx]

**S9 Appendix: Categorization of study design according to the U.S. Preventive Service Task Force (USPSTF).**

| 1. Level I: randomized control trial 2. Level II-1: controlled trials without randomization 3. Level II-2: cohort or case-control study 4. Level II-3: time series study 5. Level III: expert opinion |
| --- |
